# Non-invasive screening of breast cancer from fingertip smears—a proof of concept study

**DOI:** 10.1038/s41598-023-29036-7

**Published:** 2023-02-01

**Authors:** C. Russo, L. Wyld, M. Da Costa Aubreu, C. S. Bury, C. Heaton, L. M. Cole, S. Francese

**Affiliations:** 1 Centre for Mass Spectrometry Imaging, Biomolecular Sciences Research Centre, Sheffield Hallam University, Sheffield, UK; 2 Department of Oncology and Metabolism, University of Sheffield, Sheffield, UK; 3 Doncaster and Bassetlaw Teaching Hospitals, Doncaster, UK; 4 Department of Computing, Materials Engineering Research Centre, Sheffield Hallam University, Sheffield, UK; 5 Medicine Catapult Discovery, Manchester, UK; 6 Present Address: Department of Natural Sciences, Middlesex University, London, UK; 7 Present Address: Foster + Freeman, Evesham, UK

**Keywords:** Cancer, Biomarkers, Analytical chemistry

## Abstract

Breast cancer is a global health issue affecting 2.3 million women per year, causing death in over 600,000. Mammography (and biopsy) is the gold standard for screening and diagnosis. Whilst effective, this test exposes individuals to radiation, has limitations to its sensitivity and specificity and may cause moderate to severe discomfort. Some women may also find this test culturally unacceptable. This proof-of-concept study, combining bottom-up proteomics with Matrix Assisted Laser Desorption Ionisation Mass Spectrometry (MALDI MS) detection, explores the potential for a non-invasive technique for the early detection of breast cancer from fingertip smears. A cohort of 15 women with either benign breast disease (n = 5), early breast cancer (n = 5) or metastatic breast cancer (n = 5) were recruited from a single UK breast unit. Fingertips smears were taken from each patient and from each of the ten digits, either at the time of diagnosis or, for metastatic patients, during active treatment. A number of statistical analyses and machine learning approaches were investigated and applied to the resulting mass spectral dataset. The highest performing predictive method, a 3-class Multilayer Perceptron neural network, yielded an accuracy score of 97.8% when categorising unseen MALDI MS spectra as either the benign, early or metastatic cancer classes. These findings support the need for further research into the use of sweat deposits (in the form of fingertip smears or fingerprints) for non-invasive screening of breast cancer.

## Introduction

Breast cancer is a global health issue. In the UK 55,920 new cases of breast cancer were diagnosed annually (2016–2018), making it the most common cancer in British women^1^. The World Health Organisation (WHO) has reported that, in 2020, 2.3 million women were diagnosed with breast cancer, and approximately 30% of them had a fatal outcome, globally^2^. Furthermore, according to the WHO, breast cancer is the world’s most prevalent malignant neoplasm, given that, by the end of 2020, 7.8 million living women were registered to have received a breast cancer diagnosis in the past 5 years^2^. Rates of, and mortality from breast cancer are rising globally due to changes in population age structure and lifestyle issues. There is significant variation in outcomes, both between and within nations, due in part to variation in access to mammography, the mainstay of breast cancer screening and diagnosis^3^ and variation in access to systemic therapies.

Although mammography has facilitated earlier diagnosis and consequent improved outcomes, it is associated with exposure to radiation and physical discomfort. Mammography may be painful for many women as it requires breast compression between compression plates to enhance sensitivity. Additionally, some women find exposure of their breasts for mammography culturally unacceptable and decline screening, or delay it, even when they experience symptoms. In addition, about 10% of cancers are not visible on mammography due to high breast density^4–6^ or with certain biological subtypes such as lobular cancer^7^. In addition, in women under the age of 40, mammography has a very low sensitivity due to high breast density in this age group.

These limitations to the sensitivity of mammography may lead to delayed diagnosis which results in increased mortality rates and treatment morbidity (greater need for mastectomy, axillary clearance, chemotherapy, radiotherapy). In addition, the cost and resource issues of population screening with mammography are significant. Similarly in the diagnosis and monitoring or metastatic breast cancer, where the mainstay is contrast enhanced computed tomography (CT Scan), resources are costly and increasingly constrained.

Breast cancer diagnosis is confirmed by core needle biopsy which, again, is an invasive and painful test, and may also be technically challenging in the metastatic setting. The ability to confirm the diagnosis non-invasively would be of great value.

For the above reasons, a test that is faster, cheaper and non-invasive (and potentially more culturally acceptable), for the diagnosis of both early breast cancer and for the diagnosis and monitoring of metastatic breast cancer, would be highly desirable. It would have the potential to increase uptake of screening, reducing the stage at cancer diagnosis and therefore reducing the morbidity and mortality from early breast cancer and its treatments. In the metastatic setting it would free up NHS CT scanner capacity. It would therefore have both patient and health service benefits.

Amongst potentially suitable biological samples, breath and sweat would allow for the most non-invasive testing. Whilst the analysis of volatile organic compounds (VOCs) for cancer detection has been extensively reported in the literature, breath is rarely been the biological matrix of choice, with the first ever paper analysing VOCs in exhaled breath (breathomics) published by Sun et al. in 2007^8^. To the best of the authors' knowledge, breathomics for the specific detection of breast cancer has been reported in a handful of publications investigating classification of patients into healthy versus cancer-diagnosed and summarised by Li et al. in 2020^9^. In these studies, thermal desorption/gas chromatography/mass spectrometry^10–15^, gas chromatography/acoustic wave detection^15^ and sensor arrays^16^ were employed, showing this as a promising biological specimen to detect breast cancer.

Sweat is another interesting biological matrix which contains excreted endogenous and semi-endogenous substances as well as possible contaminants through direct skin contact or through passive diffusion from the environment^17–19^. This biological matrix is especially of toxicological interest, due to its wider detection window. Whilst of lower compositional complexity, sweat can be representative of blood through possible diffusion from the bloodstream to sweat glands and transdermal migration. In 2015, Calderon-Santiago et al. reported a study exploiting the sweat metabolome for lung cancer screening^20^. Monedeiro et al. recently published a study in which, for the first time, sweat patches were collected from healthy and diseased patients affected by different types of cancer including lung, prostate, gastric, kidney, head and neck, pancreas and colorectal cancer and lymphoma^21^. Using headspace GC–MS to analyse VOCs from these non-invasive specimen, patient classification was obtained with a 100% predictive power. To the best of the authors' knowledge there is only one publication, in the form a patent, reporting on the detection of breast cancer in sweat; here Liquid Chromatography Tandem Mass Spectrometry (LC M/MS) was used to classify healthy versus cancer-affected patients using apocrine sweat^22^. In the invention, sweat was collected from the patients’ axillae. The application of statistical analysis yielded a panel of metabolites, discriminating between the two classes of patients with a sensitivity of 97% and a specificity of 72%.

A different and potentially more advantageous approach started to emerge in 2010 when a (non-peer reviewed) report published by Belgorodsky et al.illustrated the detection of peptides and small proteins in a fingermark by Matrix Assisted Laser Desorption Ionisation Mass Spectrometry Profiling (MALDI MSP)^23^. Subsequently, in 2012, Ferguson et al.^24^ reported on the development of a MALDI MSP method specifically enabling the forensic determination of sex from fingerprints, targeting the same peptide/small protein mass range as shown by Belgorodsky et al.^23^. Ferguson et al.^24^ putatively identified some of the proteins present in these mass spectral profiles, which were detected again by Heaton et al. in 2021^25^, discovering, in addition, that some of these species had been reported as breast cancer biomarkers. These species included psoriasin (*m/z* 11,377), Dermcidin (DCD) and its C-terminal derived peptides (LEK-24, (*m/z* 2365), SSL-25 (*m/z* 2414), YDP-42 (*m/z* 4303), LEK-45 (*m/z* 4533), DCD-1 (*m/z* 4706) and DCD-1L (*m/z* 4819)). Psoriasin and dermcidin (DCD) have been previously indicated as markers for breast cancer ^26,27^. These species have been previously detected from sweat (swabs or sweat patches) using Surface-enhanced laser desorption ionization time-of-flight mass spectrometry (SELDI-TOF–MS) and reversed-phase high-pressure liquid chromatography (RP-HPLC). In 2015, Patel et al. endeavoured to optimise the MALDI MSP detection of peptides and proteins in fingerprints through in situ bottom-up proteomics, confirming the presence and identity of these potential breast cancer biomarkers^28^. This approach allowed the detection and identification of peptides/proteins of oncological interest, additional to and including dermcidin/dermcidin-derived species and psoriasin. The human cationic antimicrobial protein hCAP was identified in the study by Patel et al.^28^ and its expression levels have been correlated to tumour grade^29^. Furthermore, detection and identification of calmodulin-like protein, observed to be significantly down-regulated within invasive ductal carcinoma specimens^30^, has also been possible. Finally, within the same study, zinc α2-glycoprotein (ZAG), indicated in the literature as a possible biomarker of breast cancer differentiation^31^, has been detected.

As fingerprints are, in essence, an ordered pattern of lines made up of sweat (and any other contaminants that might have been picked up by the fingertip before contacting a surface and leaving an impression), it is hypothesised that it may be possible to detect breast cancer biomarkers from a fingertip smear (or a fingerprint in a forensic scenario). If this hypothesis is confirmed, sample collection would be even less cumbersome and most definitely less time consuming than collecting sweat patches and would indicate that, for peptide/protein biomarkers, the concentration of these species in a fingertip smear is sufficient for diagnostic purposes.

In the present study, enzymatic digestion and MALDI MSP preparation methods have been primarily optimised in both a targeted and an untargeted approach. Within the targeted approach, mass spectral profiles were evaluated for the intensity of the *m/z* signals of those proteins and peptides previously detected in fingerprints as well as of those proteins additionally indicated by the literature as potential biomarkers of breast cancer. In the untargeted approach, sample preparation protocols were evaluated according to the intensity and the range of the detected peptide ion population in the corresponding MALDI MS spectra.

The optimised methods were employed for the MALDI MSP analysis of fingertip smears collected from patients with benign breast symptoms and those with both early stage and metastatic breast cancer. These data were treated with a range of machine learning approaches to verify the hypothesis that it may be possible to detect breast cancer and classify patients from the protein content of their fingertip smears; the significance of this work lies in the perspective that if the hypothesis is verified, this method could act as a first rapid pass screening which would (i) relieve the pressure on a challenged health system (especially during and post-pandemic), (ii) contribute to saving lives due to early intervention and (iii) increase compliance due to the non-invasive nature of the test. These approaches yielded an accuracy of prediction between 62.2 and 97.8% with the latter figure being yielded by the Multi Layer Perceptron (MLP) Neural Network. These results hold the promise of a viable non-invasive screening and monitoring tool for breast cancer using fingertip smears and provide the justification for further research in a subsequent larger population study.

## Materials and methods

### Materials

ɑ-Cyano-4-hydroxycinnamic acid (CHCA), acetonitrile (ACN), trifluoroacetic acid (TFA), phosphorus red, ammonium bicarbonate, N-Octanonyl-N-methylglucamin (MEGA-8), potassium sulphate (K_2_SO_4_) and ALUGRAM SIL G/UV254 pre-coated aluminium sheets were purchased from Machery Naghel (Loughborough, UK). RapiGest^SF^ was purchased from Waters (Elstree, UK). Trypsin Gold, mass spectrometry grade (100 μg lyophilised) was purchased from Promega (Southampton, UK). Peptide calibration standard II was obtained from Bruker (Bruker Daltonics GmbH, Germany).

### Methods

#### Patient recruitment and sampling

All methods were carried out in accordance with relevant guidelines and regulations. Patients' fingertip smears were collected following full Ethics and research governance approval (IRAS ID 253281, REC reference 18/LO/1792) at a single UK breast unit. The ethics board was London—West London & GTAC Research Ethics Committee. Women were eligible for the study if they had recently been diagnosed with early breast cancer, attended breast clinic with benign pathology (breast pain, fibrocystic change, fibroadenoma etc.) or were undergoing active follow up for locally advanced (inoperable) or metastatic breast cancer. In this study, the fingertip smears of 15 patients, 5 from each category were analysed. Written informed consent was obtained from all the study participants. Details of the patients’ disease stage were recorded. Women were asked to wash their hands in a 100% ethanol solution (to remove external contaminants), dry them and wait for 15 min for new secretions to form. Each of the 3 fingertips from "sampling fingers" were then smeared across a silica-removed aluminium slide twice in the same area to obtain 3 "built up" fingertip smears. The three fingers were chosen randomly by the patient. Slides were subsequently stored in a −80 °C freezer until analysis.

#### In situ fingermarks enzymatic digestion for MALDI MS Profiling (MALDI MSP): method optimisation

For method optimisation, ungrooomed fingermarks^32^ were deposited onto aluminium slides in which the silica was removed as previously described^32^. Different digestion conditions and detergents were trialled to achieve the highest peptide ion population and the highest signal intensity. In particular: (i) trypsin concentration was either 20 or 25 μg/mL (ii) RapiGest^SF^, Mega-8 and glycerol were selected as detergents at different concentrations and individually or in mixture: RapiGest^SF^ was trialled at 0.1 or 0.2% (w/v) or in a mixture of 0.1% (w/v) with MEGA-8 2% (w/v). MEGA-8 and glycerol were used on their own as detergents only in a 2% (w/v) and 0.01% (v/v) concentration respectively (iii) enzymatic digestion time and temperature were either 2 h at 50 °C or 3 h at 37 °C; (iv) the humidity conditions during proteolysis (incubation) were explored by using either (a) a Tupperware box containing saturated K_2_SO_4_ solution (when the digestion occurred at 50 °C for 2 h), (b) a wet paper positioned at the bottom of the Tupperware box (when the digestion occurred at 37 °C for 3 h) and (c) a Tupperware box containing 50:50 H_2_O:Methanol (when the digestion occurred at 37 °C for 3 h). Table S1 summarises the digestion conditions trialled. Blank slides (controls—no fingermarks) were also digested using the same sets of conditions.

#### In situ patients' fingertip smear enzymatic digestion for MALDI MSP

Prior to proteolysis, the aluminium slides containing patients' fingertip smears were vacuum dried in order to remove the moisture generated upon removing the slides from the −80 °C temperature freezer. Three spots across the fingertip smears of 0.5 µL droplets of trypsin at concentration of 20 µg/mL in 50 mM NH_4_HCO_3_ buffer containing 0.1% (w/v) RapiGest^SF^ were deposited onto each patient’s fingertip smear and incubated in a Tupperware box containing saturated K_2_SO_4_ solution at 50 °C for 2 h.

#### Matrix deposition

After digestion a matrix solution of 10 mg/mL α-CHCA dissolved in acetonitrile (ACN)/0.5% trifluoroacetic acid (TFA) (70:30) was manually spotted (in 0.5 µL droplets) onto the localised digest areas.

#### Instrument and instrumental conditions

MALDI MS spectra were acquired using a Waters Synapt G2 HDMS mass spectrometer (Waters Corporation, Manchester, UK) equipped with a neodymium: yttrium aluminium garnet (Nd:YAG) laser operated at 1 kHz using a power adjusted to 280 arbitrary units. The instrument calibration was performed using phosphorous red. For method optimisation, MS spectra of ungroomed fingermarks were acquired, preceded by and alternated with the analysis of "blanks" in positive mode within a mass range of 600–3000 Da. All acquisitions were performed in triplicate. For the analysis of patients' fingertip smears, the instrument was calibrated using the Bruker peptide calibration standard II in positive mode over the mass range 600–2800 Da. Three spectra per fingertip smear were acquired (three technical replicates) from 3 fingertips per patient (biological replicates).

#### In silico digestion strategy for proteolysis conditions optimisation

For proteolysis conditions method optimisation, MALDI MS spectra of digested fingermarks were opened in MassLynx (Waters Corp. Manchester), converted into.txt files and imported into mMass, an open-source multiplatform mass spectrometry software^33^. Peak labelling was performed by setting the S/N threshold to 3 and deisotoping was applied. The sample peak lists were simplified by removing the matrix, matrix cluster/adducts and trypsin peaks. For putative peptide assignment, either the protein sequences of Dermcidin (P81605), Psoriasin (P31151), Calmodulin like protein 5 (Q9NZT1), Zinc α glycoprotein (P25311), Cathelicidin antimicrobial peptide (P49913), Calmodulin like protein 3 (P27482) were considered (Table S2), or a number of proteins being identified in the literature as present in fingermarks (Table S3). These sequences were preliminarily pulled out from UniProt Knowledgebase (https://www.uniprot.org/uniprot/) and linked together in silico by inserting an arbitrary sequence of 22 amino acids, RQQQQQQQQQQQQQQQQQQQQR, in between each protein and subunits, thereby generating a "master" protein sequence (Table S4). The master protein sequence was inserted into mMass and used for in silico digestion with trypsin and peptide mass fingerprinting applied to fingermarks digested with every trialled proteolysis protocol in order to determine the digestion conditions yielding the most peptides, with the highest intensity (and lowest standard deviation). For the in silico digestion, “trypsin” was selected as the enzyme; “2 missed cleavages”, “methionine oxidation”, “monoisotopic mass”, and “max charge + 1” were also selected as digestion parameters (Table S5-S7). Identifications with a relative error > 30 ppm were dismissed. Peptide identifications were verified by checking the mass accuracy of the corresponding peaks after centroiding the data. The centroid spectra were obtained by selecting the option for post-acquisition *transformation to centroid data* via automatic peak detection. Identifications were eventually only accepted if the corresponding centroid peaks had a mass accuracy within 10 ppm with respect to theoretical *m/z* values. These putatively identified peptide peaks were submitted to one final screening by checking their presence in the controls (no fingermarks) to which the same digestion protocol, acquisition and processing conditions were applied; putatively identified peaks matching "blank" peaks within 10 ppm were finally excluded from the list of putative identifications.

#### MALDI MS data processing for machine learning (ML) patient classification

Three spectra for each of the three fingertips per patients were generated totalling 135 across 15 patients. Data pre-processing of MALDI MS spectra generated from patient’s fingertip smears was carried out in MassLynx (Waters, UK). A retention time of 0.21 min was selected within the chromatogram from the start of the sample acquisition for consistency. In the resulting spectrum, Savitsky-Golay smoothing was applied with a smoothing window of ± 3, and 2 smoothing cycles. For peak annotation, the option "intensity" was selected and set to 700 a.u. as the most suitable threshold to exclude labelling of peaks with a S/N < 3. Peak centering was performed using the TOF spectrum centre function, with the minimum peak width at half height of 5, selecting the centroid top % at 80. Deisotoping was applied using the TOF transform function, using a minimum molecular mass of 700 Da, a maximum molecular mass of 2000 Da and a charge state of 1. The resulting labelled spectrum was then exported as a text file, containing two columns of data namely mass-to-charge ratio and intensity. If the intensity was below the threshold set, a value of 0 was recorded.

The remainder of the peaks were screened, and ion signals removed from the list if matching the matrix or matrix clusters/adduct and trypsin peaks within 10 ppm.

Due to the presence of cross-sample variations in *m/z* positions reported to 3 decimal places, pooling of spectra across donors initially led to a highly sparse matrix of 30,764 m*/z* peak positions across the 135 samples. However, a consistent (i.e. non-sparse) set of *m/z* values was required across all samples as the input to each supervised learning algorithm. Consequently, all *m/z* peak positions per sample were rounded to 1 decimal place, leading to a set of 5940 distinct *m/z* positions across the 135 samples. In cases where, for an individual donors' spectrum, rounding to 1 dp led to multiple intensity values at the same rounded *m/z* peak position, the maximum intensity value was taken. Any missing *m/z* positions per sample were padded with zero intensity values, leading to a dense matrix of 135 samples against 5940 m*/z* intensity values for all subsequent machine learning. A full breakdown of the processing logic steps have been made available via the supplied codebase/python Jupyter notebook deposited in the Sheffield Hallam University Research Data Archive at the link https://shurda.shu.ac.uk/id/eprint/166/ and is also illustrated graphically in Fig S1.

#### Machine learning approaches

A series of 3-class categorical supervised learning approaches have been assessed in terms of their ability to correctly predict the breast cancer status (“benign”, “early breast cancer” and “metastatic”) over the set of 135 individual *m/z* spectra associated with 15 distinct individuals (9 spectra per distinct individual, with 5 individuals per diagnosis category). The predictive performance of four distinct supervised learning algorithms has been investigated in the current study: (i) KNN (K-nearest neighbour), (ii) a decision tree, (iii) a support vector machine (SVM), and (iv) a MLP (Multilayer Perceptron). These methods were selected to represent a broad range of classical supervised learning approaches that differ in terms of complexity.

In the current study, all model algorithms were implemented in C, following the procedures defined in Russell and Norvig^34^. In the current implementations, the following hyper-parameter selections per model have been enforced throughout: (i) KNN: k = 3, linear search, Euclidean distance; (ii) decision tree: confidence factor = 0.25, number of objects per node = 2, attribute gain = top rank, entropy = classical equation; (iii) SVM: polynomial kernel function with simple logistic, c = 3.5; and (iv) MLP: learning rate = 0.001, momentum = 0.9, weights randomly initiated, single hidden layer with 150 neurons. Due to the limited available dataset size, further hyperparameter tuning was not deemed to be appropriate.

A strategy of stratified tenfold cross-validation (CV) has been applied to the dataset, where samples per diagnosis have been stratified evenly across each train and test fold such that each fold contained approximately the same percentage of samples from each diagnosis class. Furthermore, since the dataset comprises of 15 distinct individuals that each correspond to multiple distinct *m/z* spectra (3 repeats of 3 fingertip smears), each individual *m/z* spectra used for machine learning could not be assumed to be equally independent of one another. Care was therefore taken via the implemented cross validation procedure to explicitly ensure that all 9 spectra per individual were explicitly restricted to either the train or test folds (but not both) throughout. The resulting CV test folds correspond to fully unseen donors, as opposed to additional unseen spectra corresponding to donors represented in each concomitant train fold.

Due to the limited dataset size, as opposed to computing test set performance scores individually for each CV test fold and then computing the mean accuracy score across test folds, here a single out-of-sample confusion matrix per model has been derived by pooling the model inference results across each of the 10 disjoint test folds. The resulting confusion matrices comprise all 135 available samples in the dataset, however these only include predictions corresponding to unseen test folds. A categorical accuracy score has then been calculated from each out-of-sample confusion matrix, as the overall fraction of unseen samples in the dataset for which the correct diagnosis category was predicted.

Unsupervised dimensionality reduction approaches were also applied using the same prepared cross-sample matrix of 135 samples against 5940 m*/z* intensity values. 2D Principle Component Analysis (PCA) and Uniform Manifold Approximation and Projection (UMAP) algorithms have been applied, using the openly available implementations accessible via the *scikit-learn*^47^ and *umap-learn*^48^ PyPI python packages. Default algorithm parameters per package have been deemed acceptable for the purpose of the current investigation. In the case of PCA, only the first 2 principle components have been considered in order to reduce spectra to 2D. Three distinct *m/z* peak value scaling strategies have been applied prior to dimensionality reduction: (a) no scaling, (b) standardisation of each *m/z* peak position to mean = 0, standard deviation = 1 across the n = 135 dataset, and (c) min–max scaling where each m/z peak position is scaled to a fixed [0,1] domain.

## Results and discussion

In this proof-of-concept study, three categories of patients were considered, specifically those receiving a diagnosis of either "benign", early breast cancer" or "metastatic". Upon consenting to participation to the study, 3 built up fingertip smears were collected from each patient to undergo enzymatic digestion, Matrix Assisted Laser Desorption Ionisation Mass Spectrometry (MALDI MS) analysis and data treatment using ML supervised approaches.

Patel et al.^28^ had previously investigated different detergents within the enzymatic trypsin solution in order to identify that yielding the highest peptide ion population and with the highest signal intensity overall when digesting fingermarks; fingertip smears are "smudged" marks and are used in this study as its clinical nature does not require the biometric information. The conclusions of the study by Patel et al.^28^ indicated that the MEGA-8 detergent was the most highly performing when included in the enzymatic solution which was then spotted (in a 0.5% or 2% MEGA-8 concentration) or sprayed (in a 2% MEGA-8 concentration) onto the fingermark, respectively. RapiGest^SF^ however also showed promise. In their work, Patel et al.^28^, putatively identified the presence in fingermarks of proteins that have been previously indicated by the literature as biomarkers of breast cancer (depending on their up/down regulation), namely Dermcidin (P81605)^35^, Psoriasin (P31151)^36^, Zinc α glycoprotein (P25311)^37^, cathelicidin antimicrobial peptide (cAMP) (P49913)^38^, Calmodulin like protein 3 (P27482)^28,39^, additionally to other proteins only identified elsewhere such as Calmodulin like protein 5 (Q9NZT1)^40^. Using a different mass spectrometric approach and for a different purpose, Oonk et al.^41^ also identified a number of fingermarks proteins which have been taken in consideration to assess the performance of the different proteolytic conditions employed in this study. The comprehensive list of the proteins considered for the optimisation of the proteolytic conditions is reported in Table S2 and S3.

The design of experiments for this study required optimisation of the sample preparation as well as of the processing strategy. While an overview of the workflow is shown in Fig S2, below the authors systematically present and discuss the results of the experimental strategy.

Incubation conditions have considerable impact on proteolysis efficiency as optimal humidity must be reached and maintained throughout proteolysis. Work by Ly et al.^42^ investigated this aspect of proteolysis in depth and determined that the use of K_2_SO_4_ was optimal to maintain 97% humidity at 50 °C whilst avoiding condensation, also detrimental to enzymatic digestions. In their work, in addition to the use of K_2_SO_4_, the best combination of proteolytic conditions encompassed the use of trypsin at 25 μg/mL, glycerol 0.01% v/v as detergent, and an incubation time of 2 h at 50 °C (*set *(i)). The starting aim was to identify the best set of conditions yielding the highest number of literature suggested breast cancer biomarkers, in addition to any other detectable and potential protein biomarker, and with the highest sensitivity. For this reason, *Set *(i) was trialled on fingermarks and compared to other three sets of conditions in which the detergent (mixture of surfactants) was RapiGest^SF^ in 0.1% w/v concentration, as this detergent was found to be promising in the study by Patel et al.^28^. However, within these RapiGest^SF^-based conditions, *set *(ii) maintained all conditions as *set *(i) except for the concentration of trypsin trialled at 20 μg/mL as per Patel et al.^28^. *Set *(iii) maintained both the concentration of trypsin and RapiGest^SF^ as for set (ii) but increased the duration of the incubation to 3 h, decreasing the temperature to 37 °C whilst using a wet tissue to maintain humidity instead of K_2_SO_4_. Finally, set (iv) kept the same concentration of trypsin and RapiGest^SF^ and the same proteolysis duration and temperature as *set *(iii) but replaced the wet tissue with a 50/50 MeOH/water solution. These conditions are summarised in Table 1.

**Table 1:** Summary of the sets of proteolytic conditions trialled for the optimisation of the detection of protein biomarkers of breast cancer.

Sets of conditions	Trypsin concentration	Detergent	Incubation time	Temperature	Means to maintain humidity
1	25 μg/mL	Glycerol 0.01% v/v	2 h	50 °C	K_2_SO_4_
2	20 μg/mL	RapiGest^SF^ 0.1% w/v	2 h	50 °C	K_2_SO_4_
3	20 μg/mL	RapiGest^SF^ 0.1% w/v	3 h	37 °C	Wet tissue
4	20 μg/mL	RapiGest^SF^ 0.1% w/v	3 h	37 °C	50/50 MeOH/water solution

**Figure 1:**
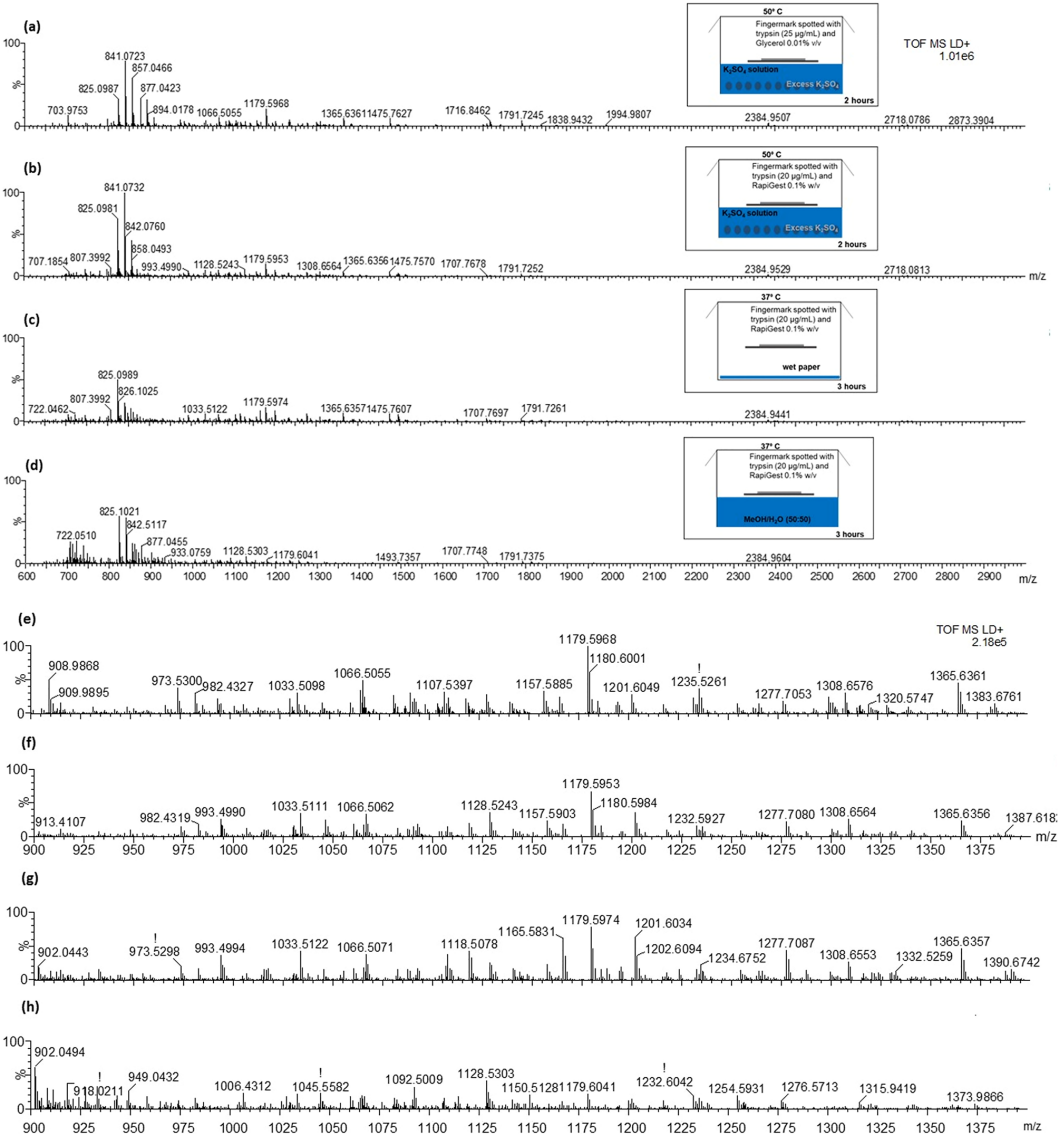
MALDI MS peptide spectra from in situ digests of ungroomed fingermarks trialled using four sets of different conditions. Panel (**a**)—*set (i):* fingermark spotted with 25 µg/mL trypsin containing 0.01% v/v glycerol and incubated in a Tupperware box containing saturated K_2_SO_4_ solution at 50 °C for 2 h; panel (**b**)—*set (ii)*: fingermark spotted using *set (i)* conditions except for trypsin used at a concentration of 20 μg/mL and RapiGest^SF^ at 0.1% replacing glycerol; panel (**c**)—*set (iii)* fingermark spotted using 20 μg/mL trypsin containing 0.1% RapiGest^SF^ and incubated at 37 °C for 3 h in a Tupperware box containing water-wet paper; panel (**d**)—*set (iv)*: fingermark spotted using same conditions as *set (iii)* but replacing the wet paper with 50:50 H_2_O:MeOH solution. Panels (**e–h**) show a zoom in the *m/z* region between 900–1400 for the spectra in the panels (**a**–**d**) respectively.

Figure 1 shows the MALDI MS spectra of fingermarks digested with the four sets of proteolytic conditions. Panels (a)–(d) illustrate the MALDI MS spectra corresponding to *sets *(i)–(iv)*,* whereas panels (e)–(h) display the corresponding zoom in the *m/z* regions 900–1400.

Table 2 reports putative peptide identifications obtained for all the four sets of proteolytic conditions trialled.

**Table 2:** Putative protein identification from in situ digests of ungroomed fingermarks spotted with *set (i):* 25 µg/mL trypsin containing 0.01% v/v glycerol and incubated in a Tupperware box containing saturated K_2_SO_4_ solution at 50 °C for 2 h; *set (ii)*: *set (i)* conditions except for trypsin used at a concentration of 20 μg/mL and RapiGest^SF^ at 0.1% replacing glycerol; *set (iii)* 20 μg/mL trypsin containing 0.1% RapiGest^SF^ and incubated at 37 °C for 3 h in Tupperware box containing water-wet paper; *set (iv)*: same conditions as *set (iii)* but replacing the wet paper with 50:50 H_2_O:MeOH solution. The asterisk indicates the proteins that are reported as breast cancer biomarkers in the literature.

Proteolytic conditions	Protein	Peptide *m/z*	Sequence	Mass accuracy (ppm)
*Set (i)* (use of K_2_S0_4_)	*Dermcidin	1128.523	ENAGEDPGLAR	−4.7
	Keratin, type I cytoskeletal 9	1065.509 1157.588 1791.724	STMQELNSR QGVDADINGLR GGSGGSYGGGGSGGGYGGGSGSR	9.1 −2.1 −1.8
	Filaggrin	1421.638	SESASRNHYGSAR	−9.4
	Sortilin-related receptor	1232.595	IEVANPDGDFR	3.6
	*Calmodulin like protein 3	1874.849	EAFSLFDKDGDGCITTR	−5.2
*Set (ii)* (use of K_2_S0_4_)	*Dermcidin	725.392 1128.524	GAVHDVK ENAGEDPGLAR	−2.6 −3.3
	Keratin, type II cytoskeletal 1	1033.511 1277.708	TLLEGEESR LALDLEIATYR	−4.7 −1.6
	Keratin, type I cytoskeletal 9	1157.590 1066.506	QGVDADINGLR FEMEQNLR	−0.5 7.1
	Filaggrin	1493.731 1513.728	QGSHHKQARDSSR QGSRHEQARDSSR	−0.5 4.3
	Desmoplakin	1107.536	AEFQEEAKR	−5.9
	Protein Shroom 3	1201.604	SLADILDPDSR	−1.9
	*Zinc a2 glycoprotein	1475.757	WEAEPVYVQRAK	−4.8
*Set (iii)* (use of wet paper)	*Dermcidin	1128.522	ENAGEDPGLAR	−5.0
	Filaggrin	1513.728	QGSRHEQARDSSR	4.4
	Keratin, type II cytoskeletal 1	1308.655	NKYEDEINKR	0.8
	Desmoplakin	1254.594 1818.990	AITGFDDPFSGK IDKQIDFRLWDLEK	−5.2 8.2
	Filamin B	1373.651	WCNEHLKCVNK	4.1
	Keratin, type I cytoskeletal10	1996.969	ELTTEIDNNIEQISSYK	−0.9
	Myosin-6	718.398	KGFPNR	−1.5
*Set (iv)* (use of 50:50 H_2_0/MeOH)	*Dermcidin	1128.530	ENAGEDPGLAR	2.0
	Keratin, type I cytoskeletal 9	1791.737 982.436	GGSGGSYGGGGSGGGYGGGSGSR FSSSGGGGGGGR	5.5 2.7
	Desmoplakin	1254.593	AITGFDDPFSGK	−5.6
	Keratin, type II cytoskeletal 1	1006.431 832.492	GGSGGGGGGSSGGR SISISVAR	1.5 3.5

The highest number of putative identifications, considering the proteins selected for this study as potential breast cancer biomarkers, were obtained when using K_2_SO_4_ to maintain humidity during proteolysis. Keratin signals were detected under all sets of conditions used; whilst expected given the biological matrix investigated, it is important to acknowledge their potential role in the ion suppression of the peptide population and subsequent impact on relevant peptide identification.

It is interesting to note that dermcidin, the antimicrobial peptide at nominal *m/z* 1128, was observed in all spectra (Fig. 1 and Table 1). Dermcidin was previously putatively detected in fingermarks by using an intact proteomic approach^24^ and its detection is particularly relevant as it has been suggested as a potential biomarker for breast cancer ^43,44^.

**Figure 2:**
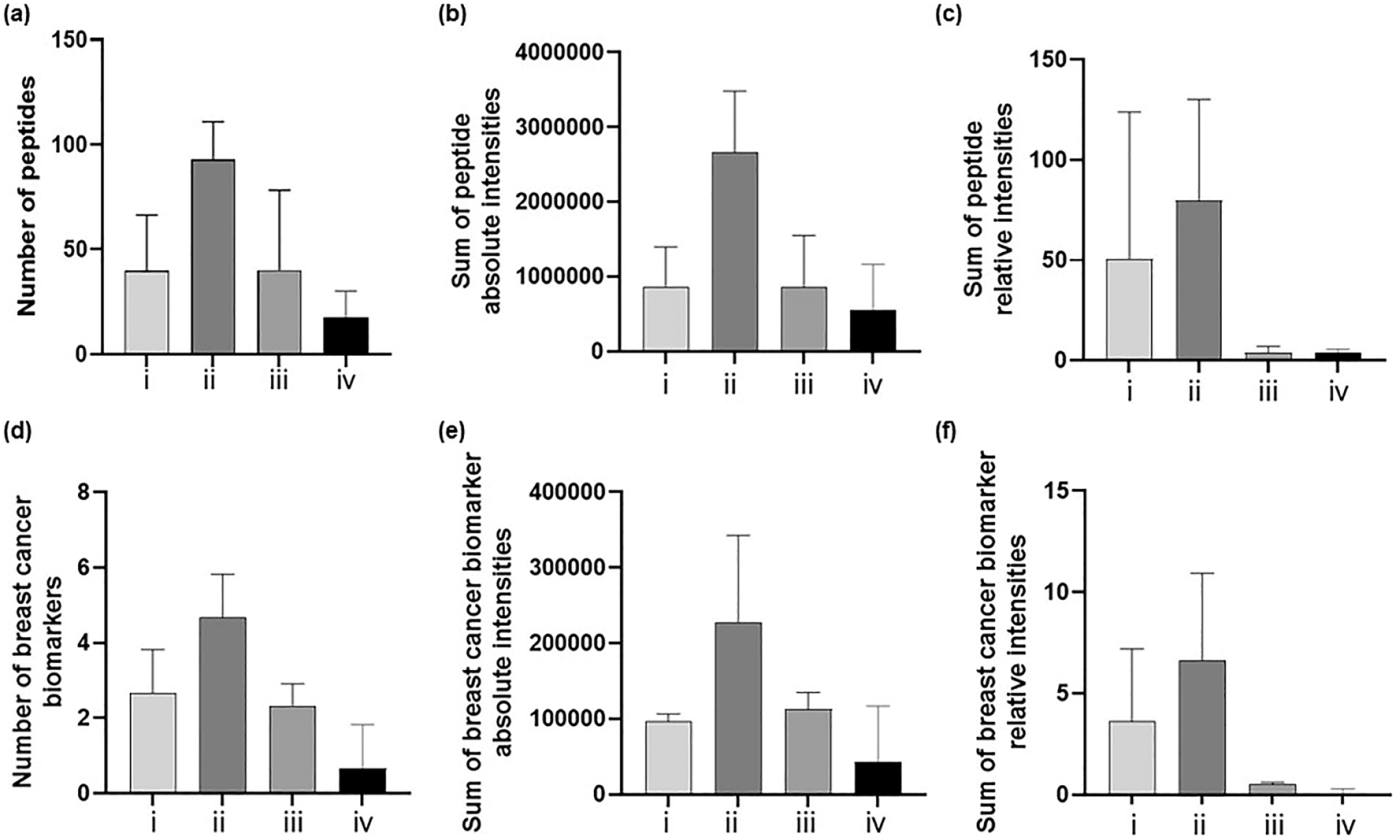
Column graph showing the number, the absolute and relative intensities of either all peptides peaks (**a**–**c**, respectively) or potential breast cancer biomarkers (**d**–**f**, respectively) present in fingermarks spotted with several digestion conditions (n = 3): (i) 25 µg/mL trypsin containing 0.01% v/v glycerol and incubated in Tupperware box containing saturated K_2_SO_4_ solution at 50 $^\circ$C for 2 h; panel *(ii)*: fingermark spotted using *set (i)* conditions except for trypsin used at a concentration of 20 μg/mL and RapiGest^SF^ at 0.1% replacing glycerol; *(iii)* fingermark spotted using 20 μg/mL trypsin containing 0.1% RapiGest^SF^ and incubated at 37 $^\circ$C for 3 h in a Tupperware box containing water wet paper; *(iv)*: fingermark spotted using same conditions as *set (iii)* but replacing the wet paper with 50:50 H_2_O:MeOH solution. For relative intensities, these were normalised with the autolysis peptide peak of trypsin at *m/z* 842.5094.

A more in-depth analysis of the performance of the four sets of conditions was undertaken by considering either the total number of peptides generated from all the proteins present in fingermarks (*instance a*) or the total number of peptides deriving from the proteins being reported as potential breast cancer biomarkers (*instance b*) (Fig. 2).

In particular, Fig. 2a–c shows the four sets of conditions (i)–(v) plotted against the total number of peptides generated (a), the sum of the peptides' absolute intensity (b) and the sum of the peptides' relative intensity (c). Figure 2 d-f follows the same schematics albeit referring to the peptides deriving from potential breast cancer protein biomarkers.

Different digestion conditions affect mass spectral quality, impacting on mass resolution, intensity and signal- to-noise (S/N). In relation to the performance of the various proteolytic conditions, (specifically referring to the means used to achieve optimal humidity and no condensation), the highest number of peptides, sum of peptides' intensity and relative intensity ratio (peptides to trypsin peaks) were achieved for both *instances a* and *b* using a saturated solution of K_2_SO_4_, with the best compromise being overall offered by the use of RapiGest^SF^ with regards to the highest number of peptides/lowest standard deviation. The same trend was observed when evaluating performance against the sum of the peptides' absolute intensity and the sum of the peptides' relative intensity for both *instances a* and *b.* The instances in which high standard deviation was observed are likely due to the manual spotting technique. A higher homogeneity of crystal distribution could be achieved using automatic spotters or possibly by combining two matrices such as alpha-cyano-4 hydroxycinnamic acid and di-hydroxybenzoic acid (CHCA-DHB).

In light of the above considerations, K_2_SO_4_ was carried forward into the next step of the method optimisation where trypsin concentration, the incubation duration and temperature were kept at 20 μg/mL, 2 h and 50 °C respectively; in these conditions, the performance of RapiGest^SF^ was compared with that of MEGA-8 2% (w/v) detergent, indicated by Patel et al.^26^ as the most effective surfactant for fingermark digestion.

**Figure 3:**
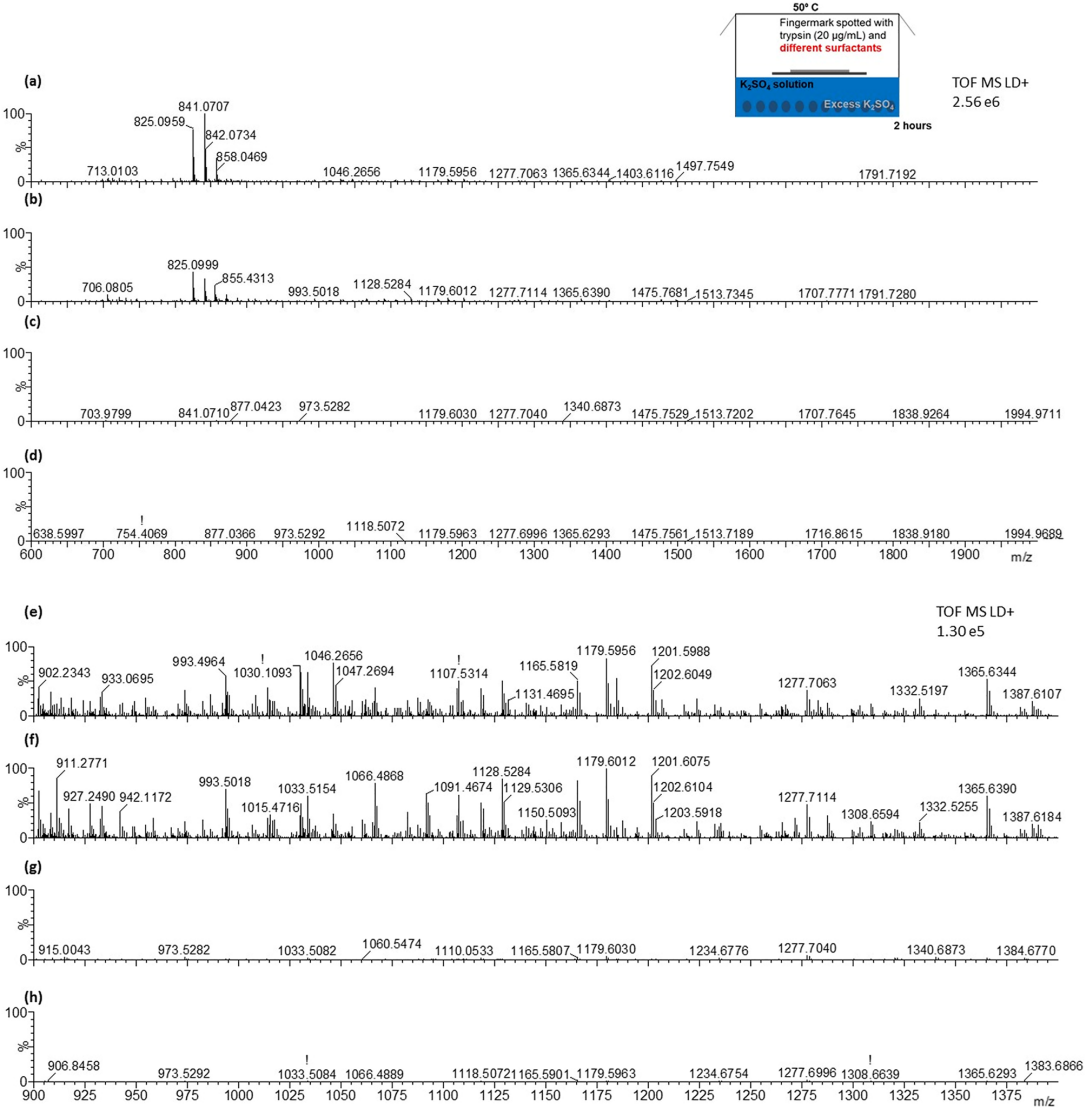
MALDI MS peptide spectra from in situ digests of ungroomed fingermarks incubated at 50 °C for 2 h in a Tupperware box containing saturated K_2_SO_4_ solution after being spotted with a 20 μg/mL trypsin solution in 50 mM NH_4_HCO_3_ buffer containing*: set (i)*—0.1% (w/v) RapiGest^SF^ (**a**), *set (ii)* 0.2% (w/v) RapiGest^SF^ (**b**), *set (iii)* 2% (w/v) MEGA-8 (**c**) or *set (iv)* a mixture of 2% (w/v) MEGA-8 and 0.1% (w/v) RapiGest^SF^ (**d**). Panels (**e**–**h**) show a zoom into the *m/z* region between 900–1400 for the spectra in the panels (**a**-**d)**, respectively.

In this second and last step of proteolysis digestion, the detergents were used as follows: set (i)—RapiGest^SF^ 0.1% (w/v); set (ii)—RapiGest^SF^ 0.2% (w/v); set (iii)—MEGA-8 2% (w/v); set (iv)—a mixture of 2% (w/v) MEGA-8 and 0.1% (w/v) RapiGest^SF^. Figure 3 shows the MALDI MS spectra for each of the further sets of conditions trialled and immediately conveys the superior performance of RapiGest^SF^, in any of the concentrations trialled over MEGA-8 or a mixture of MEGA-8 and RapiGest^SF^.

**Figure 4:**
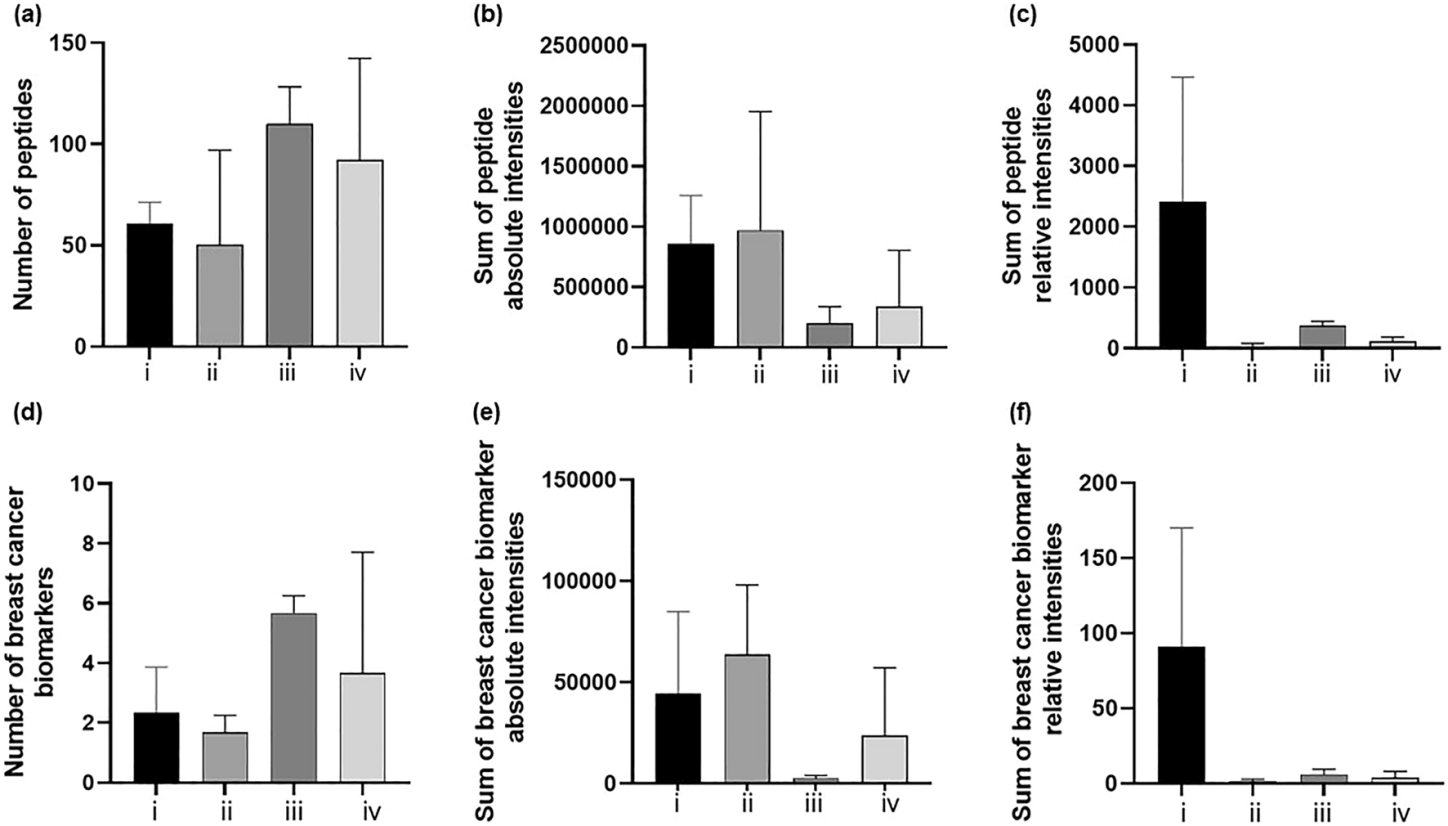
Column graph showing the number, the absolute and relative intensities of either all detected peptides peaks (**a**,**b**, **c**, respectively), or of potential breast cancer biomarkers (**d**,**e**,**f**, respectively) in fingermarks incubated for 2 h at 50 °C in Tupperware box, containing a saturated solution of K_2_SO_4_, after spotting them with a 20 μg/mL trypsin solution in 50 mM NH_4_HCO_3_ buffer containing either *Set (vi)* -RapiGest^SF^ 0.1% (w/v); *Set (ii)*—RapiGest^SF^ 0.2% (w/v); *Set* (*iii)—*MEGA-8 2% (w/v); *Set (iv)—*a mixture of 2% (w/v) MEGA-8 and 0.1% (w/v) RapiGest^SF^. For relative intensities calculation, peptides intensities were normalised against the autolysis fragment of trypsin at *m/z* 842.509.

The same in-depth performance analysis undertaken to identify the best means for optimal humidity conditions was undertaken to evaluate the most performing detergent and it is shown in Fig. 4.

With reference to the total number of peptides detected, whilst this was higher using the MEGA-8 2% (w/v) detergent versus RapiGest^SF^, the presence of several signals which were also found in blank analyses, may have contributed to relevant peptides' ion suppression (by lowering both their absolute and relative intensity). Additionally, the total absolute intensity was highest using RapiGest^SF^ 0.2% (w/v). However the relative intensity drastically dropped when the peptide ion peaks were normalised against the trypsin ion signal at *m/z* 842.509. The assessment of the proteolysis efficiency under the different conditions tested led to the conclusion that the use of Rapigest 0.1% (w/v) as detergent offered the best compromise between the abundance and intensity of peptide ions, which is very important in the context of biomarker discovery/patients classification. In terms of the total number of potential breast cancer biomarker—deriving peptides (Dermcidin (P81605), Psoriasin (P31151), Calmodulin like protein 5 (Q9NZT1), Zinc α glycoprotein (P25311 Cathelicidin antimicrobial peptide (P49913), Calmodulin like protein 3 (P27482)), a similar trend was observed, in that the highest number of peptides was again observed using MEGA-8 2% (w/v) but the sum of the peptides' absolute and relative intensity was higher for RapiGest^SF^ 0.1%(w/v).

Considering the data obtained by this 2-step method optimisation study, all patients' fingertip smears were digested by spotting a 20 μg/mL trypsin solution in 50 mM NH_4_HCO_3_ buffer containing RapiGest^SF^ 0.1% (w/v) and were subsequently incubated for 2 h at 50 °C in a silicon sealed Tupperware containing a saturated solution of K_2_SO_4_.

### Application of supervised ML approaches to MALDI MS spectra of patients' fingertip smears

MALDI MS spectra were subjected to the application of traditional supervised learning models; this approach is considered appropriate because (i) these models generate results with higher *explainability* than more recent state-of-the-art methods (e.g. deep learning), (ii) can easily operate on small datasets, such as the one collected in this proof-of-concept study, with less susceptibility to overfitting and (iii) their computational cost is relatively low^45^. Moreover, previous studies^46^ have demonstrated that such supervised learning methods can be successfully applied to high dimensional MS spectral data inputs, as is the case for the current dataset.

The four supervised learning techniques employed in this study (KNN, decision tree, SVM and MLP) were selected to cover the main supervised learning families, and as such, this strategy is meant to provide a good indication of the appropriate approach to take into a further study with a larger cohort of donors. KNN (K-nearest neighbour) is one of the simplest supervised learning algorithms and operates on the assumption that any unseen MALDI MS spectra of one cancer diagnosis class will be most similar (here via Euclidean distance) to at least one of the previously-seen training set MALDI MS spectra of the same cancer diagnosis class, than to any seen samples of other classes. The Decision tree encapsulates a mapping of input spectra to cancer diagnosis based on a learnt hierarchy of simple decision rules. It starts with a single node, which breaks down into possible outcomes. Each of these results leads to additional nodes, which branch off into other possibilities. The SVM (Support Vector Machine) is an algorithm that uses a defined kernel function to transform the input *m/z* values-containing MALDI M*S* spectra data into a higher dimensional space such that a hyperplane can be constructed to separate each cancer diagnosis class. MLP (Multilayer Perceptron) neural networks are loosely analogous to biological nervous systems, and comprise of multiple layers of fully connected nodes with learnable weights and bias terms, which, when coupled with non-linear activation functions between layers, enable complex relationships between input MALDI MS spectra and output cancer diagnosis classes to be *learnt* during training.

**Figure 5:**
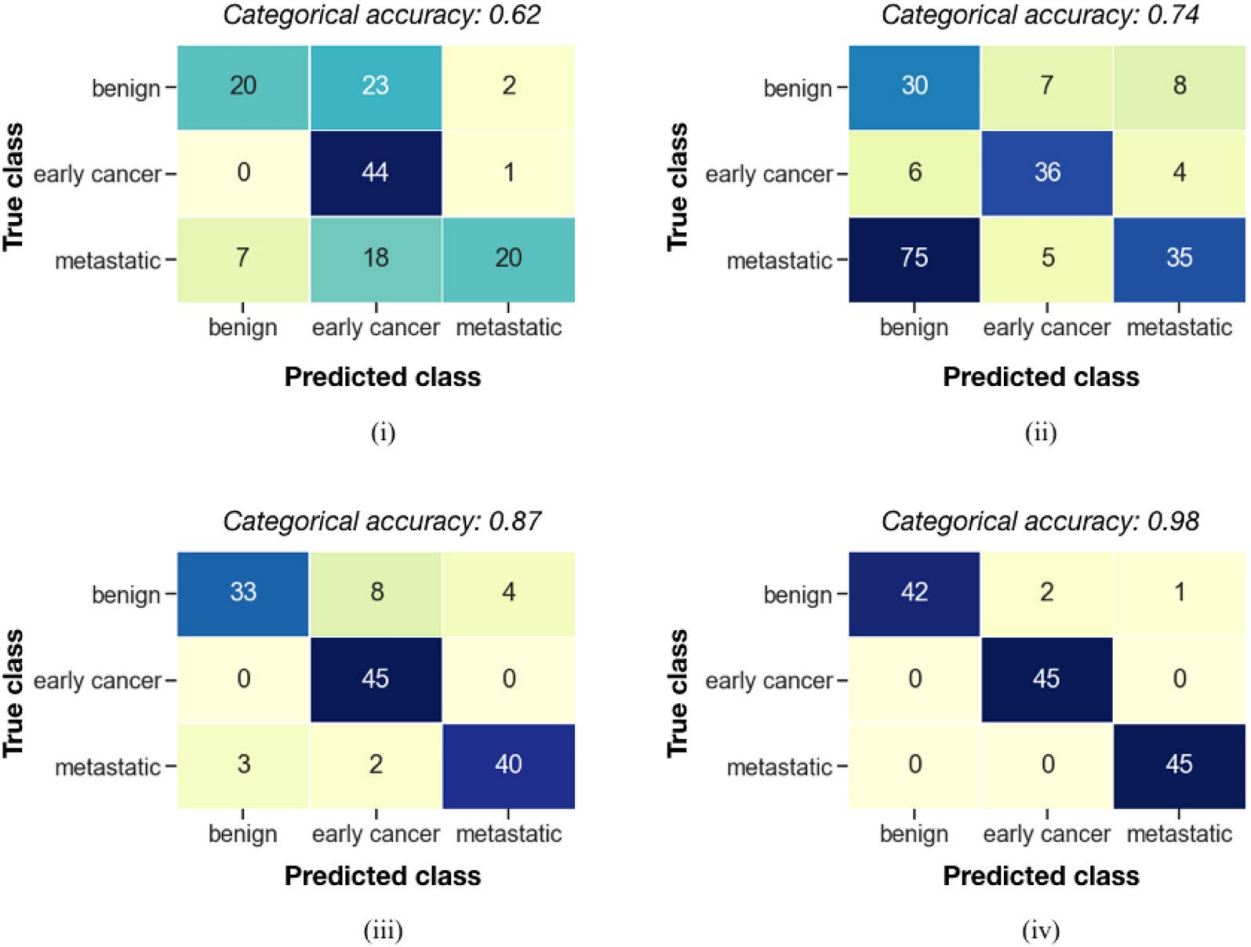
Out-of-sample confusion matrices for (i) KNN, (ii) decision tree, (iii) SVM and (iv) MLP model types. For each trialled model type, a single confusion matrix was derived from tenfold cross validation by aggregating the results for the 10 disjoint test folds. Since each individual sample features only once in a test fold, the confusion matrix features each sample exactly once, and the values per matrix sum to the total number of available samples in the full dataset. The corresponding categorical accuracy scores are reported, summarising the confusion matrix content for each model type.

Stratified tenfold cross validation was performed to assess the ability of each model architecture to suitably generalise to correct cancer diagnosis state for unseen samples. Figure 5 illustrates the resulting out-of-sample confusion matrices of correctly and wrongly classified samples per model, derived by aggregating the results across the 10 disjoint test folds during cross validation.

**Figure 6:**
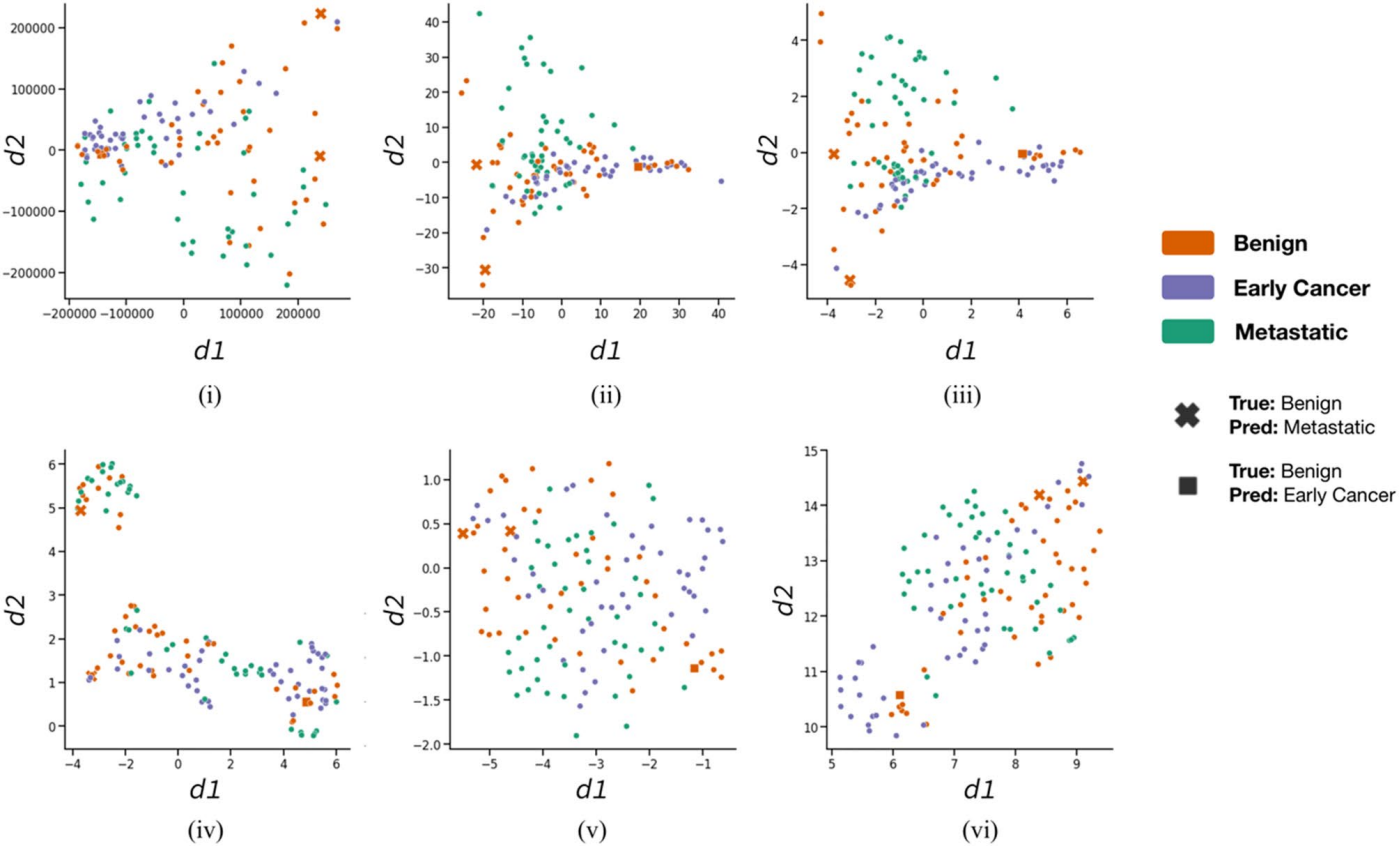
Dimensionality reduction of spectra to 2D using (i-iii) PCA and (iv-vi) UMAP algorithms. Results are illustrated for three independent spectra scaling approaches, applied prior to dimensionality reduction: (i, iv) no additional scaling, (ii, v) standardisation of each *m/z* peak position to mean = 0, standard deviation = 1 across the n = 135 dataset, and (iii, vi) min–max scaling where each m/z peak position is scaled to a fixed [0,1] domain. In each subplot, each scatter point corresponds to an individual sample in the n = 135 dataset, coloured by cancer diagnosis state. The three benign samples that were misclassified by the MLP model as “metastatic” and “early cancer” have been highlighted as cross and square markers, respectively.

All investigated model types displayed above random performance for the cancer diagnosis task, and the qualitative ranking of reported categorical accuracy scores correlated with algorithm complexity. As expected, KNN being the simplest model exhibited the lowest categorical accuracy across cancer classes (~ 62.2%), most notably with a high rate of metastatic and benign cancer samples being misclassified as early cancer. Such a high false classification rate is unacceptable for viable usage in a clinical setting, but provides a baseline for evaluating other predictive methods. In contrast to KNN, the other methods attained consistently higher overall accuracy scores, and appeared to be more robust to the high rate of misclassification of the early cancer pathology affecting the KNN model. Overall this is indicative of the expected superior learning ability of these methods compared to KNN. Across methods, the best categorical accuracy score was attained by the trialled MLP approach (~ 97.8%), with this method leading to no early or metastatic cancer samples being misclassified under the current *k*-fold cross validation regime. Besides MLP, all other model types resulted in multiple metastatic samples in the dataset being falsely classified as benign; such cases of cancer samples being incorrectly classified as benign are highly undesirable for clinical usage. In the case of MLP, an unsupervised dimensionality reduction analysis of spectra across the n = 135 dataset (Fig. 6) did not provide a clear explanation for the 3 misclassified spectra; namely the misclassified samples did not consistently appear to be constituents of well-defined other-class clusters (or nearest neighbours to other-class spectra) across the trialled reduction algorithms (PCA, UMAP) and spectra pre-processing strategies. In conclusion, the current investigation has presented initial findings on the potential viability of a series of classical supervised machine learning methods for the clinical application of breast cancer diagnosis prediction via usage of MALDI MS collected by non-invasive methods. Despite low availability to model training data in current study, with an overall dataset size of 135 MALDI MS spectra collected from 15 individual donors, a cross validation strategy has been implemented which explicitly accounts for replicate spectra per donor and enables the evaluation of each machine learning method’s ability to generalise to unseen patient samples; its application only yielded three samples incorrectly classified.

## Conclusions

This proof-of-concept study has shown the significant potential for a novel, rapid, non-invasive and sensitive screening methodology to detect the molecular signatures of breast cancer from a simple swipe of a fingertip. The combined proteomics-MALDI MS approach to the detection of protein biomarkers and the use of classical supervised machine learning methods for data treatment hold the promise for a novel screening method of this type of pathology, especially for women with metastatic breast cancer for whom a tissue diagnosis may be challenging and imaging may be equivocal.

Out of the trialled methods, the MLP neural network architecture exhibited the highest ability to appropriately classify unseen breast cancer samples (test accuracy score ~ 97.8% under *k*-fold cross validation), and importantly, for the current dataset, led to no life-threatening metastatic cancer samples being falsely flagged as benign. Such model behaviour is close to ideal for clinical usage, however, is likely to be strongly dependent on the limited dataset size available in the current study (135 MALDI MS spectra collected from 15 individual donors). Nonetheless, clear evidence has been presented that such supervised machine learning methods could be leveraged to infer breast cancer diagnosis for unseen patients when provided with input MALDI MS data.

This method may also be important in monitoring disease progression for women on chemotherapy for metastatic or locally advanced disease and in whom serial CT scans or MRI are presently required. Therefore, having a non-invasive, non-imaging based test as an adjunct to current diagnostic modalities would reduce rates of mis-diagnosis and improve early diagnosis and cure rates. The pain-free nature of the test would likely increase screening and survival rates; for a negative response, it also eliminates the risk of un-necessary radiation exposure.

Both these benefits and these initial findings motivate the need for an upscaled data collection over a larger breast cancer donor cohort, in order to robustly assess model generalisation across a patient set of increased diversity.

## Supplementary Information

Supplementary Information 1.

Supplementary Information 2.

Supplementary Information 3.

Supplementary Information 4.

Supplementary Information 5.

Supplementary Information 6.

Supplementary Information 7.

Supplementary Information 8.

## Data Availability

Data and supporting information are openly available from the Sheffield Hallam University Research Data Archive at the link https://shurda.shu.ac.uk/id/eprint/166/. Mass Spectra data have been supplied in three formats: raw spectra (in the proprietary Waters Corp Synapt G2 HDMS format), exported as text files (.txt) and as the associated processed dataset that has been used for downsteam ML (.csv). In addition, a Jupyter notebook containing all the data pre-processing steps, and also the subsequent undersupervised learning analysis.
