# Supplementary material for: Non-invasive screening of breast cancer from fingertip smears—a proof of concept study
Source: Sci Rep. 2023 Feb 1;13:1868. doi: 10.1038/s41598-023-29036-7 (PMC9892587; doi:10.1038/s41598-023-29036-7)
Supplement: Supplementary file 1 — Supplementary Information 1. [file 41598_2023_29036_MOESM1_ESM.docx]

**Supplementary material**


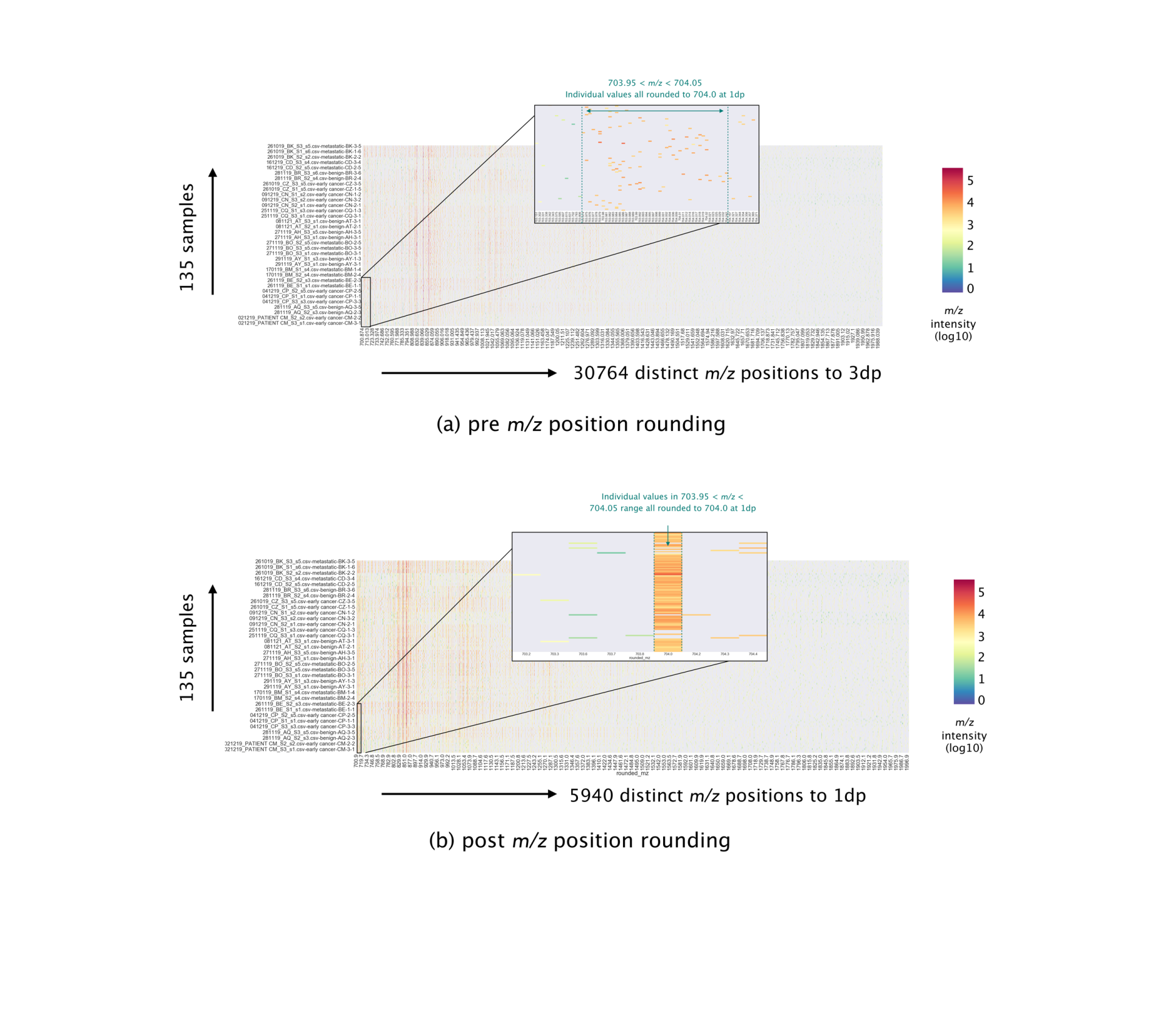


**Figure S1**: Donor sample ID versus *m/z* intensity matrices for all distinct recorded *m/z* positions (a) pre *m/z* position rounding (i.e. to 3dp) and (b) post *m/z* position rounding to 1dp. In both cases, each individual element position in each matrix corresponds to the reported intensity value for a particular donor sample (vertical axis) at a particular distinct *m/z* position (horizontal axis). Available numerical *m/z* intensity values are illustrated by a spectrum colour scheme derived from the logarithm (base 10) of the values; conversely empty/non-assigned matrix positions are coloured in grey. In (a) there are 30764 distinct m/z positions reported to 3 decimal places; as illustrated by the magnified insert in (a), there is evident cross-sample variations in *m/z* positions to 3dp, resulting in a highly sparse matrix in which most matrix positions are not assigned numerical values. For classical machine learning approaches, such high data sparsity is non-favourable, primarily since such ML methods aim to learn repeated trends across conserved sets features (the *m/z* intensity values here). In (b) the effect of rounding all *m/z* peak positions per donor sample to 1 decimal place has been illustrated, which here led to a set of 5940 distinct *m/z* positions across the 135 samples. The equivalent magnified insert in (b) illustrated how the spread of recorded *m/z* positions between 703.95 and 704.05 are condensed into a singular band of intensity values at 704.0 to 1dp. For downstream machine learning applications, this now enables these cross-sample intensity values to be directly compared and treated as one single input feature to machine learning. It should be noted that since the rounding of *m/z* positions to 1dp could, in principle, lead to multiple intensity values at the same rounded *m/z* peak position for an individual donors' spectrum, in these cases, explicit logic was implemented to compute the maximum intensity value whenever multiple intensity values were binned in the same 1dp *m/z* peak position. As highlighted previously in this figure caption, a consistent (i.e. fully non-sparse) set of *m/z* values was required across all samples as the input to each supervised learning algorithm. As illustrated in (b) 1dp rounding does not achieve this here (since non-assigned matrix positions are still prevalent in grey). Consequently, as a final processing step prior to downstream machine learning, all remaining non-assigned *m/z* positions per sample in (b) were padded with zero intensity values, leading to a dense matrix of 135 samples against 5940 *m/z* intensity values. A full breakdown of the processing logic steps are available via the supplied codebase/python Jupyter notebook deposited in the Sheffield Hallam University Research Data Archive at the [link](https://shurda.shu.ac.uk/id/eprint/163) https://shurda.shu.ac.uk/id/eprint/166/


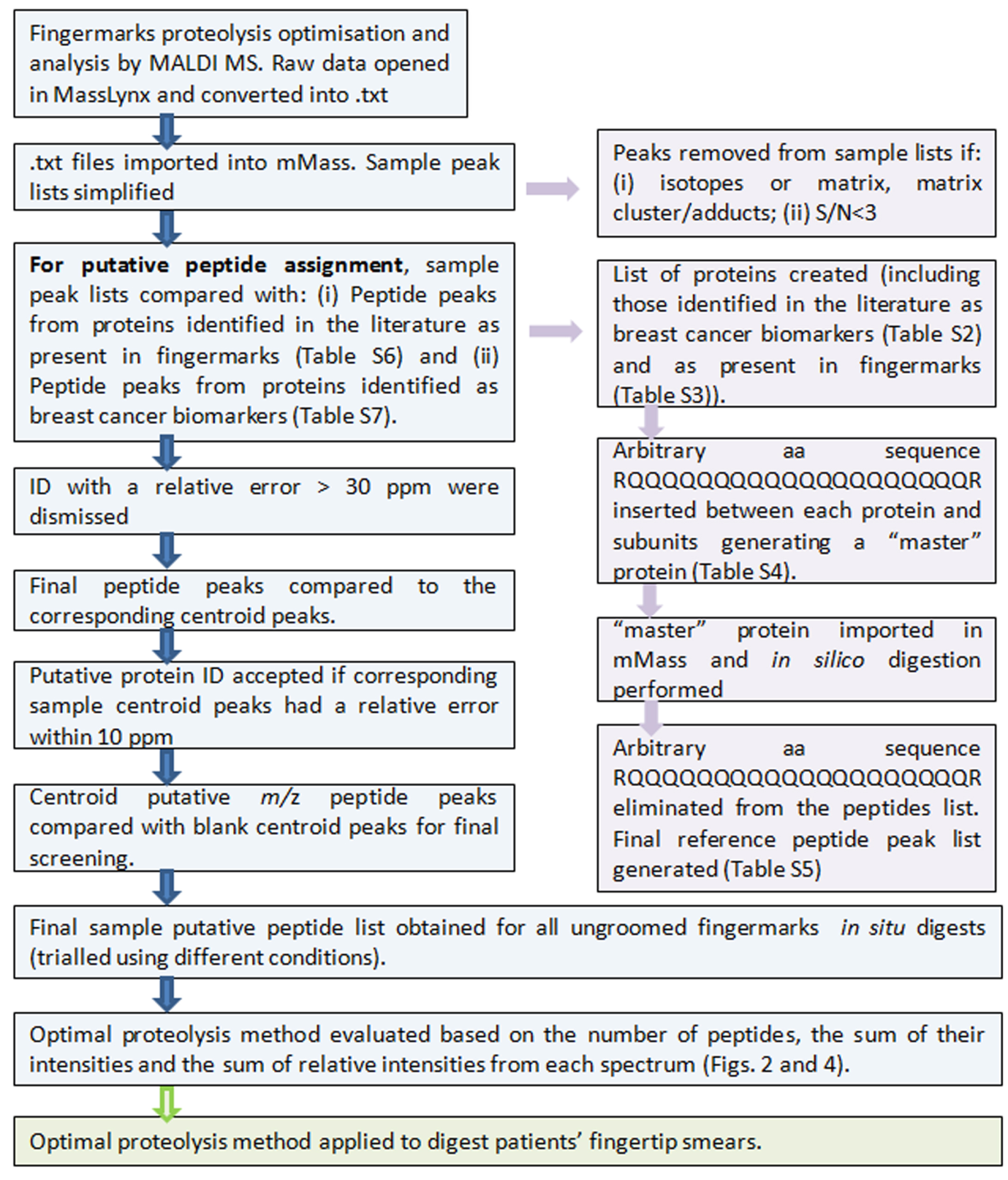


**Figure S2.** Flowchart of the methodology leading to the optimal data acquisition from patients' fingertip smears
