# Supplementary material for: Non-invasive screening of breast cancer from fingertip smears—a proof of concept study
Source: Sci Rep. 2023 Feb 1;13:1868. doi: 10.1038/s41598-023-29036-7 (PMC9892587; doi:10.1038/s41598-023-29036-7)
Supplement: Supplementary file 2 — Supplementary Information 2. [file 41598_2023_29036_MOESM2_ESM.docx]

| **Trypsin solution** | **Digestion incubation** | **Temperature and duration** |
| --- | --- | --- |
| - 25 μg/mL in 50 mM NH_4_HCO_3;_ 0.01% glycerol - 20 μg/mL in 50 mM NH_4_HCO_3;_ 0.1% RapiGest^SF^ - 20 μg/mL in 50 mM NH_4_HCO_3_; 2% MEGA-8 - 20 μg/mL in 50 mM NH_4_HCO_3_; mixture of 2% MEGA-8 and 0.1% RapiGest SF - 20 μg/mL in in 50mM NH_4_HCO_3_; 0.2% RapiGest^SF^ | Saturated K_2_SO_4_ solution | 50⁰C; 2hours |
| 20 μg/mL in 50 mM NH_4_HCO_3;_ 0.1% RapiGest^SF^ | - 50% H2O: 50% MeOH - Wet paper | 37⁰C; 3hours |

**Table S1**. Summary of the protocols employed for in situ digestion of fingermarks.
